# Processing speed dysfunction is associated with functional corticostriatal circuit alterations in childhood epilepsy with centrotemporal spikes: a PET and fMRI study

**DOI:** 10.1007/s00259-022-05740-w

**Published:** 2022-02-24

**Authors:** Yuting Li, Teng Zhang, Jianhua Feng, Shufang Qian, Shuang Wu, Rui Zhou, Jing Wang, Guo Sa, Xiawan Wang, Lina Li, Feng Chen, Hong Yang, Hong Zhang, Mei Tian

**Affiliations:** 1 Department of Nuclear Medicine and Medical PET Center, The Second Hospital of Zhejiang University School of Medicine, 88 Jiefang Road, Hangzhou, 310009 Zhejiang China; 2 Institute of Nuclear Medicine and Molecular Imaging of Zhejiang University, Hangzhou, China; 3 Key Laboratory for Biomedical Engineering of Ministry of Education, Zhejiang University, Hangzhou, China; 4 Key Laboratory of Medical Molecular Imaging of Zhejiang Province, Hangzhou, China; 5 Department of Pediatrics, The Second Hospital of Zhejiang University School of Medicine, Hangzhou, China; 6 Department of Radiology, The First Hospital of Zhejiang University School of Medicine, Hangzhou, China; 7 College of Medical Imaging, Shanxi Medical University, Taiyuan, China; 8 The College of Biomedical Engineering and Instrument Science, Zhejiang University, Hangzhou, China

**Keywords:** Epilepsy, Positron emission tomography (PET), Resting-state functional magnetic resonance imaging (Rs-fMRI), Processing speed

## Abstract

**Purpose:**

Epilepsy with centrotemporal spikes (ECTS) is the most common epilepsy syndrome in children and usually presents with cognitive dysfunctions. However, little is known about the processing speed dysfunction and the associated neuroimaging mechanism in ECTS. This study aims to investigate the brain functional abnormality of processing speed dysfunction in ECTS patients by using the ^18^F-fluorodeoxyglucose (^18^F-FDG) positron emission tomography (PET) and resting-state functional magnetic resonance imaging (rs-fMRI).

**Methods:**

This prospective study recruited twenty-eight ECTS patients who underwent the ^18^F-FDG PET, rs-fMRI, and neuropsychological examinations. Twenty children with extracranial tumors were included as PET controls, and 20 healthy children were recruited as MRI controls. The PET image analysis investigated glucose metabolism by determining standardized uptake value ratio (SUVR). The MRI image analysis explored abnormal functional connectivity (FC) within the cortical–striatal circuit through network-based statistical (NBS) analysis. Correlation analysis was performed to explore the relationship between SUVR, FC, and processing speed index (PSI).

**Results:**

Compared with healthy controls, ECTS patients showed normal intelligence quotient but significantly decreased PSI (*P* = 0.04). PET analysis showed significantly decreased SUVRs within bilateral caudate, putamen, pallidum, left NAc, right rostral middle frontal gyrus, and frontal pole of ECTS patients (*P* < 0.05). Rs-fMRI analysis showed absolute values of 20 FCs were significantly decreased in ECTS patients compared with MRI controls, which connected 16 distinct ROIs. The average SUVR of right caudate and the average of 20 FCs were positively correlated with PSI in ECTS patients (*P* = 0.034 and *P* = 0.005, respectively).

**Conclusion:**

This study indicated that ECTS patients presented significantly reduced PSI, which is closely associated with decreased SUVR and FC of cortical–striatal circuit. Caudate played an important role in processing speed dysfunction.

**Clinical trial registration:**

NCT04954729; registered on July 8, 2021, public site, https://clinicaltrials.gov/ct2/show/NCT04954729

**Supplementary Information:**

The online version contains supplementary material available at 10.1007/s00259-022-05740-w.

## Introduction

Epilepsy with centrotemporal spikes (ECTS) is the most common focal epilepsy syndrome in children, accounting for 15–20% of pediatric epilepsy [1]. Although ECTS was originally treated as “benign” due to its relatively low seizure frequency [2], current consensus has reached that ECTS is not benign and can lead to delayed cognitive and behavioral maturation [3, 4]. Language and working memory impairments have been widely investigated in ECTS patients [3, 5]; however, little is known about processing speed dysfunction. Processing speed is a key cognitive ability that measures the capacity of decision-making and visual and auditory information processing [6]. Specially, processing speed dysfunction may weaken the learning efficiency of ECTS patients, who are usually school-age children [7]. In order to provide rational bases to improve processing speed, it is necessary to investigate processing speed dysfunction and its associated brain functional abnormality in ECTS patients.

The cortical–striatal circuit provides a physiologic basis for processing speed, as described in previous studies of dementia, trauma, and schizophrenia [8–10]. In this circuit, information is transferred from the cerebral cortex to basal ganglia through the striatum, which can be anatomically divided into caudate, putamen, and nucleus accumbens (NAc), primarily associated with cognitive, motor, and motivational processes, respectively [11]. Particularly, the large amount of interictal epileptic discharges in ECTS patients may disrupt local neuronal activity and functional connectivity of the circuit [12]. Local neuronal activity changes within the circuit can be assessed by glucose metabolic rate using ^18^F-fluorodeoxyglucose (^18^F-FDG) positron emission tomography (PET) molecular imaging [13]. On the other hand, the functional connectivity alternations can be measured by synchronous fluctuations of blood oxygen level–dependent (BOLD) using resting-state functional magnetic resonance imaging (rs-fMRI) [14]. Furthermore, the combination of PET and rs-fMRI can provide a rich description of functional abnormality for the in-depth investigation of processing speed dysfunction in ECTS patients.

Therefore, in this study, we hypothesize that epileptic discharges of ECTS patients disrupt corticostriatal connections, which is associated with impairments in processing speed. Thus, this study aimed to investigate the glucose metabolism and functional connectivity of cortical–striatal circuit and the relationship with processing speed dysfunction in ECTS patients.

## Materials and methods

### Participants

**Fig. 1:**
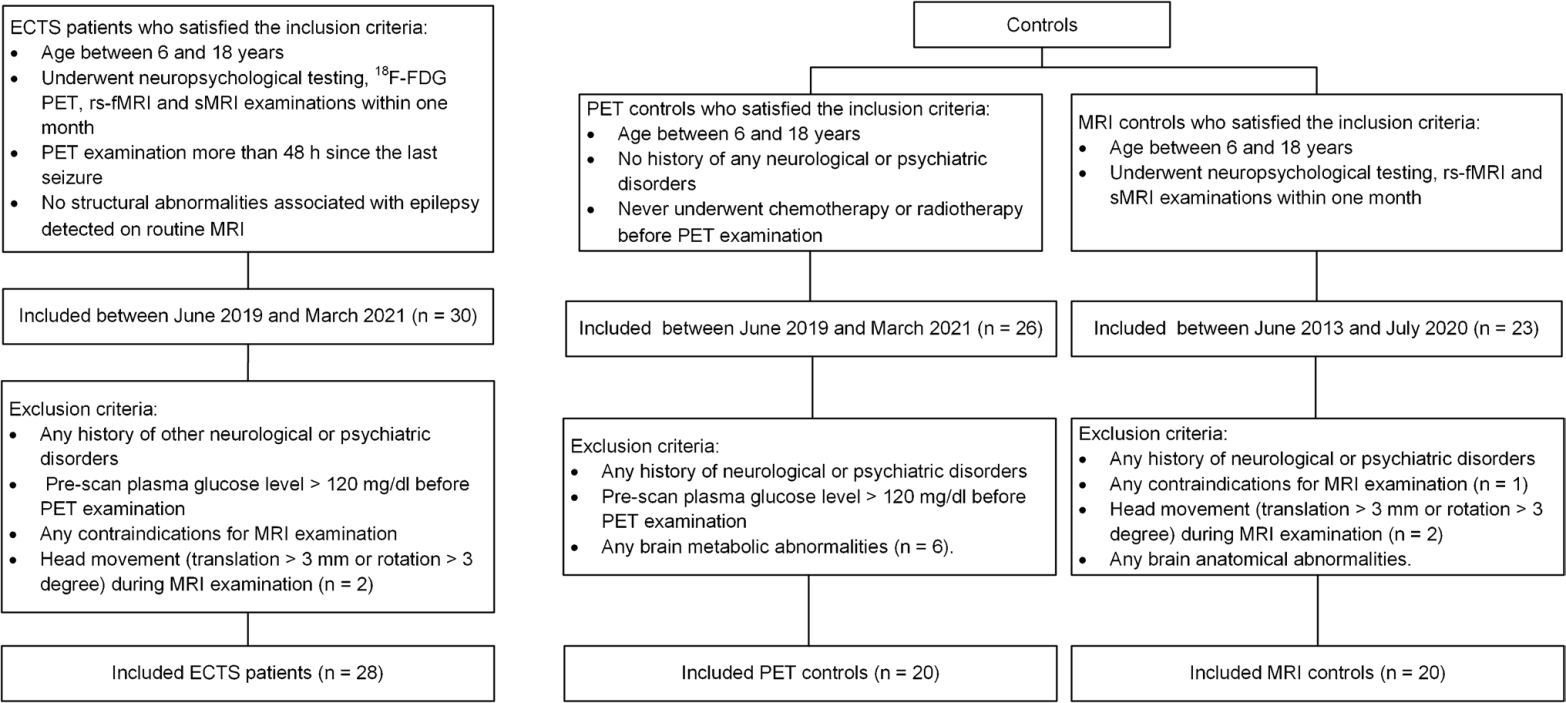
Flowchart of participants’ selection. ECTS, epilepsy with centrotemporal spikes

Thirty patients (13 girls and 17 boys, mean age = 9.8 ± 2.6 years) diagnosed as ECTS in our hospital were recruited between June 2019 and March 2021. All patients underwent neuropsychological testing, ^18^F-FDG PET/CT, rs-fMRI, and structural MRI (sMRI) examinations within 1 month. The inclusion criteria were as follows: (a) clinical diagnosed as ECTS by an experienced pediatric neurologist, including history of tonic–clonic convulsive nocturnal seizures or simple partial seizures during the waking hours and EEG-confirmed classic centrotemporal spikes [15]; (b) age between 6 and 18 years; (c) ^18^F-FDG PET examination more than 48 h since the last seizure; and (d) no structural abnormalities associated with epilepsy detected on routine MRI. The exclusion criteria included (a) any history of other neurological or psychiatric disorders; (b) pre-scan plasma glucose level > 120 mg/dl before ^18^F-FDG PET/CT examination [16]; (c) any contraindications for MRI examination; and (d) head movement (translation > 3 mm or rotation > 3 degree) during MRI examination [12]. Two patients were excluded due to movement during resting-state scan. At last, twenty-eight patients were thus included in this study (Fig. 1).

Twenty-three age- and gender- matched healthy children (7 girls and 16 boys, mean age = 10.6 ± 2.2 years) were enrolled as controls in MRI analysis. The controls underwent neuropsychological testing, 3 T sMRI, and rs-fMRI examination but except PET examination. Two children were excluded due to movement during resting-state scan, and one child was excluded due to MRI contraindications. To avoid unnecessary radiation, another dataset of 26 age- and gender-matched children with extracranial tumors (10 girls and 16 boys, mean age = 10.4 ± 2.6 years) was included as controls in PET image analysis [17]. The PET controls had no history of any neurological or psychiatric disorders and never underwent chemotherapy or radiotherapy before PET examination. PET and routine MRI images of these children were carefully reviewed by two experienced physicians to exclude subjects with metabolic or anatomical abnormalities. Discordant results were reviewed by the two physicians to reach a consensus. Six children were excluded due to metabolic abnormalities. In total, twenty healthy children were included in MRI image analysis, and twenty children with extracranial tumors were included in PET image analysis (Fig. 1). The institutional review board approved the present study, and written informed consent was obtained from all participants.

### Neuropsychological assessment

All ECTS patients and MRI controls underwent a comprehensive neuropsychological examination to test cognitive performance. Intelligence was assessed by the Wechsler Children’s Intelligence Scale Fourth Edition (WISC-IV), which includes four scales: verbal comprehension index (VCI), perceptual reasoning index (PRI), working memory index (WMI), and processing speed index (PSI) [18].

### Image acquisition

PET images were acquired by a PET/CT scanner (Biograph mCT, Siemens Medical Solutions). All patients fasted for at least 6 h before injection with a standard dose of ^18^F-FDG (3.7 MBq/kg). Then a 5-min brain scan was carried out approximately 40 min after injection. PET data were obtained after overnight withdrawal of treatment (at least 12 h) for ECTS patients.

MRI images were acquired by a 3 T MRI scanner (GE Medical Systems, Signa HDX, USA) with an 8-channel high-resolution head coil. Earplugs and foam padding were used to limit head movement and reduce scanner noise for the subjects. 3D T1-weighted sMRI images were first acquired using fast spoiled gradient recalled (3D-FSPGR) sequence with the following parameters: 150 sagittal slices (thickness/gap = 1/0 mm), repetition time (TR) = 7 ms, echo time (TE) = 2.85 ms, flip angle = 8 degree, and field of view (FOV) = 256 × 256 mm^2^. Rs-fMRI scans were acquired using echo-planar images (EPIs) with the following parameters: 29 slices (thickness/gap = 5/0 mm), TR = 2000 ms, TE = 30 ms, FOV = 240 × 240 mm^2^, and flip angle = 90 degree. During MRI scanning, subjects were instructed to lie still with eyes closed and not to fall asleep.

### PET image processing

^18^F-FDG PET images were preprocessed by Statistical Parametric Mapping (SPM8; https://www.fil.ion.ucl.ac.uk/spm), FSL (https://fsl.fmrib.ox.ac.uk/fsl/fslwiki) [19], and FreeSurfer (https://surfer.nmr.mgh.harvard.edu/) [20]. All ^18^F-FDG PET images of ECTS patients and controls were spatially normalized to an in-house pediatric ^18^F-FDG PET template, followed by Gaussian smoothing with 6-mm full-width half-maximum (FWHM) [21]. Standardized uptake value ratio (SUVR) was determined to normalize intensities by using cerebellum gray matter as the reference region [22].

In order to validate the hypothesis that the cortical–striatal circuit is the physiologic basis for processing speed in ECTS patients, SPM analysis was first performed to detect all potential metabolic changes across whole brain regions. *P* value < 0.05 and cluster size > 100 were used as thresholds. Then regional analysis was carried out on region of interests (ROIs) which contained at least 100 continuous voxels of SPM clusters, according to the pediatric PET Desikan–Killiany atlas. The ROIs were also thresholded by gray matter (GM) probability > 60% to reduce the influence of white matter and cerebrospinal fluid retention [23]. The weighted average of regional SUVRs was compared between ECTS patients and controls by two-sample *t* test, and false positive rate (FDR) was performed to correct multiple comparison. At last, Pearson’s correlation between PSI and mean SUVR was investigated for ECTS patients. Construction of pediatric PET template, Desikan–Killiany atlas, and GM probability map was described in the Supplementary Material S1.

### MRI processing

#### Data preprocessing

3 T sMRI scans were preprocessed by FreeSurfer (https://surfer.nmr.mgh.harvard.edu/) [20]. The preprocessing steps included the removal of non-brain tissues, bias field correction, gray-white matter segmentation, gray-white matter boundary tessellation, and tissue segmentation [24]. Whole-brain ROIs were derived in each subject’s native space according to the Desikan–Killiany atlas. Rs-fMRI images were preprocessed by DPARSF (https://www.restfmri.net) [25] and FSL [19] (https://fsl.fmrib.ox.ac.uk/fsl/fslwiki). The first 10 images were excluded to reduce magnetization disequilibrium, followed by slice timing correction and head motion correction. The preprocessed sMRI was aligned to corresponding BOLD image by rigid registration using FSL, along with transformation of ROIs according to Desikan–Killiany atlas. BOLD images were then spatial smoothed using a 6-mm FWHM Gaussian kernel, followed by linear detrending and temporal band pass filtering (0.01–0.08 Hz) to remove low- and high-frequency noises. Nuisance covariates were regressed out to improve the signal-to-noise ratio using the Friston-24 head motion parameters, as well as white matter signal and corticospinal fluid signal. The mean framewise displacements (FDs) of each subject were calculated, and fames with FD > 0.2 mm were removed to reduce the influence of head movement (scrubbing).

### Functional network analysis

PSI-related functional connectivities (FCs) were compared between ECTS patients and controls by network analysis. For each subject, time series of each node were extracted from preprocessed BOLD images using DPARSF. Pearson correlation was calculated between time series of all ROI pairs according to Desikan–Killiany atlas, and then Fisher’s *Z* transformed as interregional FC. To reduce the influence of spurious interregional connectivity, one-sample *t* test was performed for each FC in ECTS patients and controls, followed by FDR to correct multiple comparison [26]. Pearson’s correlation was estimated between PSI and FCs with *P* > 0.05 in either ECTS patients or controls, and then the PSI-related FCs were used in following network-based statistic (NBS) analysis. The NBS analysis involved three major steps [27]. The difference of mean FC between ECTS patients and controls was first determined. Then, nonparametric permutation test was performed by randomly re-allocating all subjects into two groups with the same sizes as ECTS and control groups. In each permutation, the mean FC difference was determined to estimate permutation distributions, from which *P* values were derived according to ranking of differences. At last, multiple comparison was corrected by suprathreshold cluster size test, which extracted the largest connected component from significant connections (*P* < 0.05) in each permutation. The top 5% largest component size was used as the threshold. Relationships between NBS subnetworks, i.e., the largest connected components, were also investigated.

### Statistical analysis

Statistical analyses of the clinical information were performed using SPSS 22.0 (https://www-01.ibm.com/software/analytics/spss). Continuous variables were presented as means ± standard deviations (SD), and categorical variables were presented as numbers (proportions). Significant differences between patients and controls were compared using two-sample *t* test for continuous variables and the chi-square test (χ2) for categorical variables, respectively. Stepwise multivariate linear regression analysis was performed to evaluate the effects of clinical factors (gender, age at scan, age at seizure onset, seizure duration, the time of AED treatment, antiepileptic drugs, and EEG at diagnosis) on VCI, PRI, WMI, PSI, and full-scale intelligence quotient (FSIQ) in ECTS patients. The influence of seizure duration, time of AED treatment, and AED treatment types (AED-naïve, monotherapy, and polytherapy) on PSI, glucose metabolism, and functional connectivity were also investigated (Supplementary Material S2, S3). Pearson correlation was used in the correlation analysis of ECTS patients. *P* < 0.05 was considered statistically significant.

## Results

### Clinical information

**Fig. 2:**
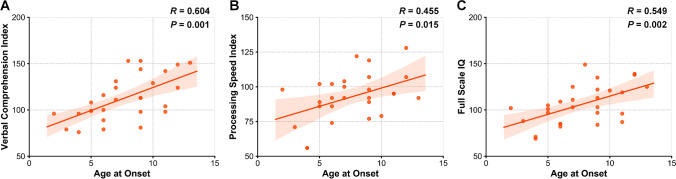
The relationship between age at onset and IQ of ECTS patients. Age at onset was positively correlated with **A** VCI; **B** PSI; and **C** full-scale IQ. ECTS, epilepsy with centrotemporal spikes; VCI, verbal comprehension index; PSI, processing speed index; IQ, intelligence quotient

The clinical characteristics of ECTS patients and controls were presented in Table 1. The PSI of ECTS patients was significantly decreased than controls (*P* = 0.04), and no significant differences in VCI (*P* = 0.70), PRI (*P* = 0.32), WMI (*P* = 0.94), and FSIQ (*P* = 0.26) were found between ECTS patients and healthy controls. In multivariate linear regression analysis, age at seizure onset was the only significant factor for VCI (*P* = 0.001), PSI (*P* = 0.015), and FSIQ (*P* = 0.002) (Fig. 2).

**Table 1:** Clinical Information of ECTS patients and controls

Clinical characteristics	ECTS (*n* = 28)	Controls (*n* = 20)	*P* value
Gender			0.58
Female	12	7	
Male	16	13	
Age at scan (years)	9.96 ± 2.62	10.75 ± 2.07	0.27
Age at seizure onset (years)	7.68 ± 2.92	N/A	––
Seizure duration (months)	30.18 ± 26.26	N/A	––
The time of AED treatment (months)	19.39 ± 26.09	N/A	––
Antiepileptic drug			––
Monotherapy	11	N/A	
Polytherapy	10	N/A	
None	7	N/A	
Lateralization on EEG at diagnosis			––
Left	5	N/A	
Right	8	N/A	
Bilateral	15	N/A	
Verbal comprehension index	112.71 ± 23.95	114.95 ± 16.38	0.70
Perceptual reasoning index	107.00 ± 17.86	111.15 ± 9.83	0.32
Working memory index	99.92 ± 17.82	100.30 ± 16.13	0.94
Processing speed index	92.96 ± 16.88	102.55 ± 13.32	0.04
Full-scale intelligence quotient	105.54 ± 21.12	110.95 ± 12.10	0.26

*ECTS*, epilepsy with centrotemporal spikes; *EEG*, electroencephalogram

In patients with ECTS, 39.3% (*n* = 11) of patients underwent monotherapy, and 35.7% (*n* = 10) underwent polytherapy. The most frequently used AED was oxcarbazepine (OXC, *n* = 13), followed by levetiracetam (LEV, *n* = 12), valproate (VPA, *n* = 5), and lamotrigine (LTG, *n* = 1) (Table 2). The seizure duration (*P* = 0.763), time of AED treatment (*P* = 0.555), and AED treatment types showed no significant influence (*P* = 0.789) on PSI.

**Table 2:** Information of antiepileptic drugs in patients

Treatment	ECTS (*n* = 28)
AED-naïve	7
AED treatment	
OXC	5
VPA	3
LEV	3
OXC + LEV	8
VPA + LTG	1
VPA + LEV	1

*OXC*, oxcarbazepine; *VPA*, valproate; *LEV*, levetiracetam; *LTG*, lamotrigine

### Metabolic analysis

**Fig. 3:**
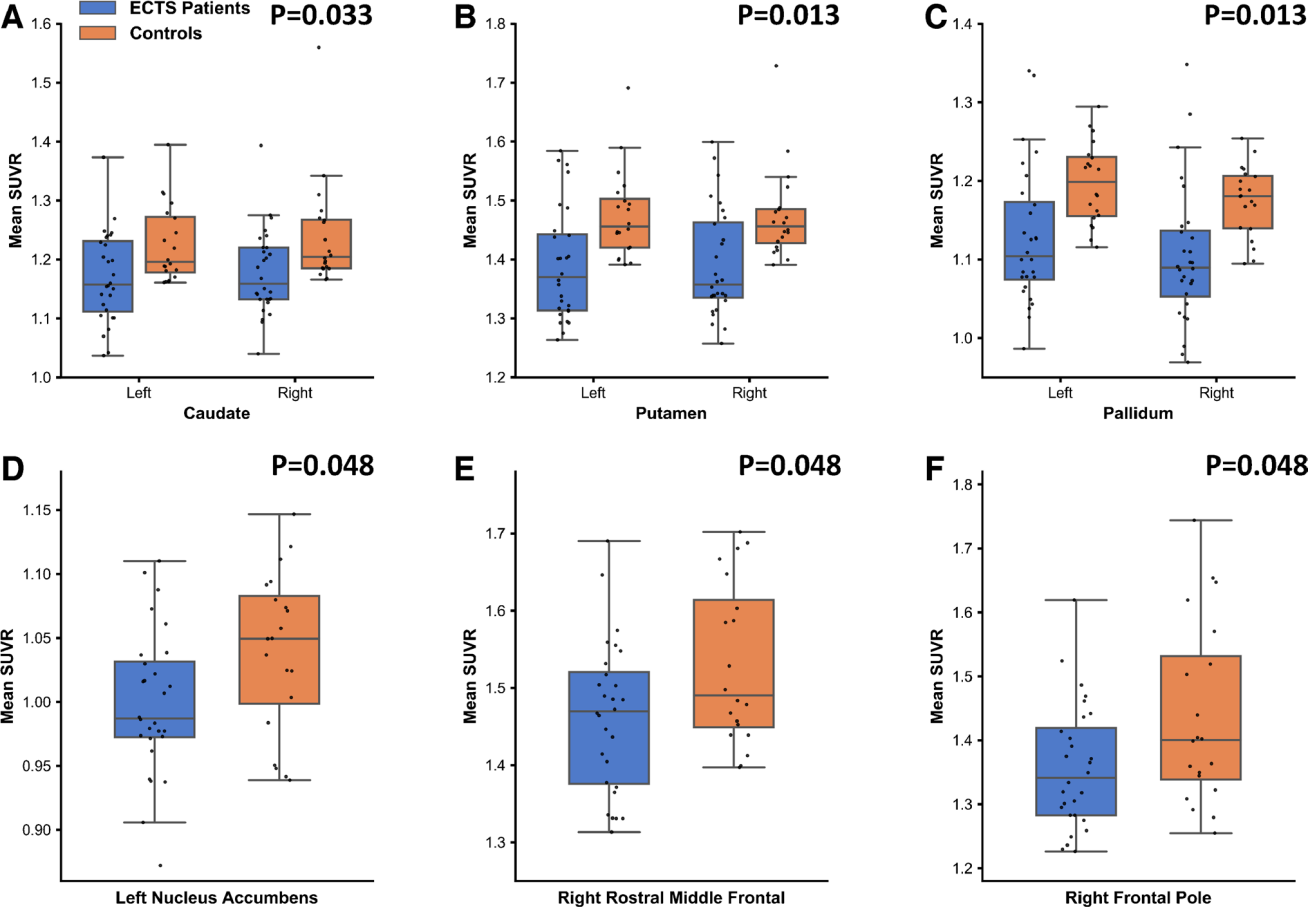
Significantly decreased SUVRs in ECTS patients compared with controls. **A** bilateral caudate; **B** bilateral putamen; **C** bilateral pallidum; **D** left nucleus accumbens; **E** right rostral middle frontal; **F** right frontal pole

**Fig. 4:**
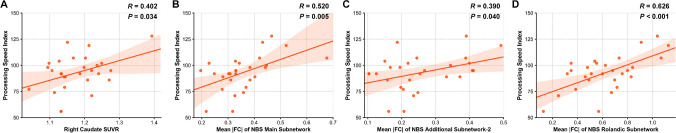
The relationship between PSI, regional SUVR, and FC in ECTS patients. PSI was positively correlated with **A** average SUVR of right caudate; **B** mean absolute FC within NBS main subnetwork; **C** mean absolute FC within NBS additional subnetwork-2; and **D** mean absolute FC between bilateral rolandic areas. ECTS, epilepsy with centrotemporal spikes; PSI, processing speed index; SUVR, standardized uptake value ratio; FC, functional connectivity

Compared with the PET controls, ECTS patients showed distributed hypo-metabolism in the bilateral caudate, putamen, pallidum, lateral orbitofrontal gyri, rostral middle frontal gyri, superior frontal gyri, left thalamus, NAc, right caudal middle frontal gyrus, and right frontal pole (Supplementary Fig. S1). Regional analysis was performed on these ROIs (Table 3), and significantly decreased regional metabolism was found in bilateral caudate, putamen, pallidum, left NAc, right rostral middle frontal gyrus, and frontal pole of ECTS patients (*P* < 0.05, FDR corrected) (Fig. 3). Pearson’s correlation analysis showed that only the SUVR of the right caudate was positively related with PSI (*P* = 0.034) (Fig. 4A). The seizure duration, time of AED treatment, and AED treatment types showed no significant influence on regional SUVR (Supplementary Material S2, S3).

**Table 3:** Comparison of regional metabolism between ECTS patients and controls

ROI	ECTS (*n* = 28)	Controls (*n* = 20)	*P* value uncorrected	*P* value FDR corrected
Left thalamus	1.162	1.143	0.321	0.341
Left caudate	1.170	1.226	0.012	0.033*
Right caudate	1.178	1.239	0.011	0.033*
Left putamen	1.390	1.475	0.002	0.013*
Right putamen	1.392	1.473	0.002	0.013*
Left pallidum	1.128	1.197	0.003	0.013*
Right pallidum	1.104	1.175	0.002	0.013*
Left accumbens	0.999	1.040	0.023	0.048*
Left lateral orbitofrontal	1.286	1.322	0.141	0.176
Left rostral middle frontal	1.499	1.546	0.128	0.176
Left superior frontal	1.382	1.412	0.191	0.216
Right caudal middle frontal	1.511	1.562	0.072	0.111
Right lateral orbitofrontal	1.255	1.287	0.145	0.176
Right precentral	1.371	1.393	0.352	0.352
Right rostral middle frontal	1.462	1.531	0.025	0.048*
Right superior frontal	1.377	1.423	0.055	0.094
Right frontal pole	1.358	1.439	0.024	0.048*

*ECTS*, epilepsy with centrotemporal spikes; *FDR*, false discovery rate; *P < 0.05

### Disrupted network connectivity of ECTS

**Fig. 5:**
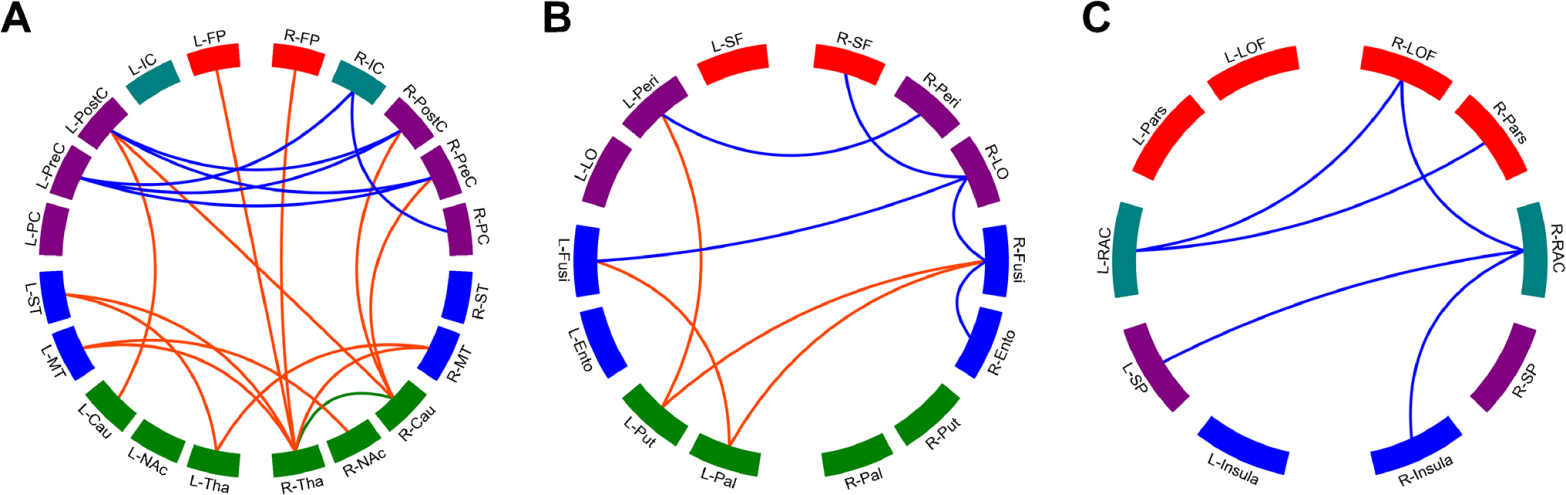
Network-based statistic analysis results between controls and ECTS patients. Cau, caudate; Ento, entorhinal; FP, frontal pole; Fusi, fusiform; IC, isthmus cingulate; insula, insula; LO, lateral occipital; LOF, lateral orbitofrontal; MT, middle temporal; NAc, nucleus accumbens; Pal, pallidum; Pars, pars triangularis; PC, precuneus; Peri, pericalcarine; PostC, postcentral; PreC, precentral; Put, putamen; RAC, rostral anterior cingulate; SF, superior frontal; SP, superior parietal; ST, superior temporal; Tha, thalamus

The adjacency matrix of ECTS patients and MRI controls were shown in Supplementary Fig. S2. NBS analysis showed that absolute values of 20 FCs were significantly decreased in ECTS patients compared with MRI controls, which connected 16 distinct ROIs (NBS cluster size threshold = 12) (Table 4). This main subnetwork involved a pathway from rolandic areas to the caudate, thalamus, and the cortical regions, which is part of the cortical–striatal circuit (Fig. 5A). If suprathreshold cluster size test for NBS multiple test correction was avoided, two additional small PSI-related subnetworks could also be found (Fig. 5B–C). The two small subnetworks connected 15 ROIs through 16 FCs (Supplementary Table S1). If PSI-unrelated FCs were also included, connections could be found among the main subnetwork and additional subnetworks (Supplementary Fig. S3). Moreover, ROIs within the main subnetwork was more densely connected than the additional subnetworks.

**Table 4:** Comparison of PSI-related FC between ECTS patients and controls

Functional connectivity		ECTS (*n* = 28)	Controls (*n* = 20)	*P* value
Region 1	Region 2			
Left thalamus	Left superior temporal	− 0.101	− 0.274	0.034
Left thalamus	Right middle temporal	− 0.116	− 0.292	0.024
Left caudate	Left postcentral	− 0.185	− 0.409	0.004
Right thalamus	Right caudate	0.487	0.844	0.006
Right thalamus	Left middle temporal	− 0.138	− 0.301	0.026
Right thalamus	Left superior temporal	− 0.110	− 0.316	0.009
Right thalamus	Left frontal pole	− 0.021	− 0.198	0.007
Right thalamus	Right middle temporal	− 0.081	− 0.274	0.029
Right thalamus	Right frontal pole	0.000	− 0.155	0.047
Right caudate	Left postcentral	− 0.196	− 0.422	0.005
Right caudate	Right postcentral	− 0.146	− 0.383	0.004
Right caudate	Right precentral	− 0.141	− 0.310	0.029
Left nucleus accumbens	Left middle temporal	0.049	− 0.123	0.009
Left postcentral	Right postcentral	0.863	1.138	0.011
Left postcentral	Right precentral	0.456	0.767	0.002
Left precentral	Right isthmus cingulate	− 0.229	− 0.342	0.018
Left precentral	Right postcentral	0.530	0.736	0.034
Left precentral	Right precentral	0.623	0.837	0.028
Left precuneus	Right isthmus cingulate	0.509	0.725	0.019
Right isthmus cingulate	Right precuneus	0.516	0.767	0.001

*ECTS*, epilepsy with centrotemporal spikes; *FC*, functional connectivity; *PSI*, processing speed index

Absolute values of FCs within the main NBS subnetwork (*P* = 0.005) and the additional subnetwork-2 (*P* = 0.040) were positively correlated with PSI in ECTS patients (Fig. 4B–C). However, the correlation between that within additional subnetwork-1 and PSI was not significant in ECTS patients (*P* = 0.090). In particular, absolute values of FCs between bilateral rolandic areas (precentral and postcentral gyri) were positively correlated with PSI in ECTS patients (*P* < 0.001) (Fig. 4D). The seizure duration, time of AED treatment, and AED treatment types showed no significant influence on PSI-related FCs (Supplementary Tables S2 and S3).

## Discussion

In this study, we investigated the processing speed dysfunction in ECTS patients using ^18^F-FDG PET and rs-fMRI. Our study showed that the reduced PSI was associated with the decreased SUVR and FC of cortical–striatal circuit in ECTS patients. Particularly, the caudate played an important role in processing speed dysfunction. To the best of our knowledge, this is the first study to investigate functional neuroimaging biomarkers of processing speed dysfunction in ECTS patients.

Most neuroimaging studies concentrated on cognitive impairments of language, attention, and working memory in ECTS patients [1, 3]; however, only one study investigated processing speed [21]. Our study, together with the previous study, showed that processing speed could be impaired in patients with ECTS [28]. The functional abnormality of processing speed could be associated with the large amount of epileptic charges during interictal period, despite relatively infrequent seizures in patients with ECTS [12]. In our study, the main PSI-related NBS subnetwork involved FCs between bilateral rolandic areas. Moreover, the decreased absolute FC between bilateral rolandic areas was significantly correlated with reduced PSI in ECTS patients. It could suggest that epileptic discharges originated from rolandic areas result in processing speed dysfunction of ECTS patients.

Our findings indicated that functional abnormality of cortical–striatal circuit was associated with processing speed impairment in ECTS patients. The cortical–striatal circuit is the neural pathway connecting cortical regions and basal ganglia to mediate motor, cognitive, and behavioral functions [11]. In this pathway, the bilateral caudate, putamen, pallidum, left NAc, right rostral middle frontal gyrus, and frontal pole showed significantly decreased glucose metabolism in ECTS patients, indicating reduced neuronal activity in these regions [13]. In addition, SUVR in the caudate was positively correlated with PSI. Moreover, the PSI-related NBS main subnetwork involved FCs between the bilateral rolandic areas and FCs from the rolandic areas to the caudate, the thalamus, and then the frontal and temporal lobes. It may indicate how the epileptic discharges influence the cortical–striatal circuit. The caudate could be directly influenced by the epileptic discharges originated from the rolandic areas. This finding is consistent with a previous finding that caudate is particularly vulnerable to pathological factors due to its topological centrality [29]. The decreased SUVR and FC of caudate could therefore suggest that the caudate is a primary structure of processing speed dysfunction in ECTS patients, in consistence with a previous study of processing speed in traumatic brain injury [30].

The cortical–striatal circuit from NBS analysis also involved cortical regions including the frontal pole (FP) and the superior temporal (ST) and middle temporal (MT) gyri. The FP played an import role in decision processing [31], and ST and MT gyri were considered to be associated with speech processing [32]. The main NBS subnetwork also involved precuneus and isthmus cingulate, impairments of which were related to slowed processing speed [31, 33]. Moreover, there were significantly decreased connections between NBS main and additional subnetworks, which were not correlated with PSI and thus removed from NBS analysis. Therefore, the two NBS additional subnetworks may be branches of the cortical–striatal circuit in the main subnetwork. The cortical regions in the two branches, including the fusiform, pericalcarine, lateral orbitofrontal, rostral anterior cingulate, and superior parietal, were also considered to be associated with processing speed [33–37].

In this study, ECTS patients were treated with the second-generation AEDs, including OXC, LEV, VPA, and LTG. These new AEDs had very little side effects on cognition, and most of them could even protect processing speed ability [38–43]. The group comparison showed no significant differences in PSI among AED-naïve patients and patients who underwent monotherapy and polytherapy. Therefore, the processing speed impairment could be resulted from epileptic discharges rather than AED treatment. The processing speed depends on various functional abilities, such as attention, planning, and visuospatial and auditory skills [6]. These functions are supported by complex brain network that provides the physiological basis for information processing [44]. In this study, the decreased FCs were connecting to regions related to processing speed and formed a subnetwork associated with decreased PSI. It indicated that network deficit may be the pathological basis of processing speed dysfunction in ECTS, similar to other neuropsychological disorders [30, 45]. As pediatric brains are highly plastic, processing speed can be improved like other cognitive abilities. Our findings could provide rational bases to improve processing speed of ECTS patients, such as physical exercise [46], brain training game [47], and transcranial stimulation [48].

On the other hand, AED could still influence glucose metabolism and functional connectivities. VPA could reduce whole-brain glucose metabolism; however, this reduction is very slight at a negligible level [40]. Patients taking LEV showed increased glucose metabolism in the bilateral caudate, frontal lobes, and left parietal lobe [40], and those taking LTG showed reduced glucose metabolism in the thalamus and basal ganglia [49]. In a previous fMRI study, ECTS patients who underwent AED treatment showed decreased FC in default network, while those who did not take AEDs showed increased FC in default network and motion-related networks [16]. Similarly, a combined EEG-fMRI study showed increased whole-brain FC after withdrawal of AEDs in focal epilepsy [50]. Although group comparison showed no significant difference in regional SUVR and FC among AED-naïve patients and patients who underwent monotherapy and polytherapy, the potential influences cannot be totally avoided and formed a major limitation of our study. Similar to previous studies, the AED treatment was specialized by an experienced physician according to patient seizure condition in this study [17, 51]. Future works may consider to recruit more AED-naïve patients to validate our findings.

## Conclusion

This study indicated that ECTS patients presented significantly reduced PSI, which is closely associated with decreased SUVR and FC of the cortical–striatal circuit. Caudate played an important role in processing speed dysfunction.

## Supplementary Information

Below is the link to the electronic supplementary material.

Supplementary file1 (DOCX 1606 KB)
