# Supplementary material for: Processing speed dysfunction is associated with functional corticostriatal circuit alterations in childhood epilepsy with centrotemporal spikes: a PET and fMRI study
Source: Eur J Nucl Med Mol Imaging. 2022 Feb 24;49(9):3186–96. doi: 10.1007/s00259-022-05740-w (PMC9250469; doi:10.1007/s00259-022-05740-w)
Supplement: Supplementary file 1 — Supplementary file1 (DOCX 1606 KB) [file 259_2022_5740_MOESM1_ESM.docx]

**SUPPLEMENTARY MATERIAL**

**S1. Construction of pediatric ^18^F-FDG PET template, Desikan-Killinay atlas and gray matter probability map**

The pediatric ^18^F-FDG PET template was constructed by an iterative registration-averaging optimization approach, as described in our previous study [21]. Briefly, this optimization approach repeatedly registered pediatric ^18^F-FDG PET images of controls into a temporary template, and updated transformations by residual errors. The average of all registered images were used as PET template. The iterative optimization was terminated when no difference was found between templates of two consecutive iterations. This procedure can minimize metabolic and morphologic differences between pediatric PET images and template.

T1-weighted structural MRI (sMRI) images of controls were preprocessed by Freesurfer (<https://surfer.nmr.mgh.harvard.edu/>), including removal of non-brain tissue, bias field correction, gray-white matter segmentation, gray-white matter boundary tessellation, and tissue segmentation. The preprocessed sMRI images were then coregistered with corresponding PET images by EPI rigid registration, and parcellations according to Desikan-Killiany atlas were transformed to individual PET space by nearest-neighbor interpolation. Then, the PET images were spatially normalized to the pediatric ^18^F-FDG PET template by affine and SyN registration by using advanced normalization tools (<http://stnava.github.io/ANTs/>). Individual parcellations were transformed to the common pediatric PET template space by affine and deformation fields from spatial normalization. For each voxel in the PET template, a 4-mm full-width-half maximum Gaussian kernel was applied to summarize probabilities of ROIs within Gaussian kernel radius. Voxel was then labeled as that of ROI with maximum summed probability according to Desikan-Killiany atlas. At last, this pediatric Desikan-Killiany atlas was then modified manually by an experienced radiologist.

The gray matter (GM) probabilities were obtained by segmenting sMRI by using Statistical Parametric Mapping (<https://www.fil.ion.ucl.ac.uk/spm>). The GM probabilities were transformed to corresponding PET image by rigid registration, and then the pediatric ^18^F-FDG PET template space by affine and SyN registration. Average of all GM probabilities were regarded as GM probability map for pediatric ^18^F-FDG PET template, and voxels with GM probability > 60% were used as GM mask in our study.

**S2: Influence of seizure duration and AEDs treatment time**

In order to investigate the effect of seizure duration on attention network, patients with ECTS were divided into two subgroups according to seizure duration: subgroup-1 with the seizure duration ≥ 2 years (n = 15) and subgroup-2 with the seizure duration < 2 years (n = 13). The ECTS patients were also grouped according to AED treatment time by using 1 year as threshold, and the same partition was obtained as those according to seizure duration, possibly due an average interval time of 11 months between seizure onset and treatment. The thresholds of seizure duration = 2 year and treatment time = 1 year were arbitrarily selected to balance subgroup sizes. Network-based statistics (NBS) analysis was then performed between the two subgroups, and no significant subnetwork was found (cluster size threshold = 11) except four isolated functional connectivities (**Supplementary Table S2**). Therefore the effect of seizure duration and AED treatment time on attention network may be negligible in this study.

**S3: Influence of Antiepileptic Drugs**

In patients with ECTS, 39.3% (n=11) of patients underwent monotherapy, and 35.7% (n=10) underwent polytherapy. The most frequently used AED was oxcarbazepine (OXC, n=13), followed by levetiracetam (LEV, n=12), valproate (VPA, n=5), and lamotrigine (LTG, n=1). We compared PSI and SUVR among AED-naïve patients, patients who underwent monotherapy and polytherapy by using Kruskal-Wallis test. No significant difference was found in PSI (*P* = 0.789), and neither in regional SUVRs (*P* > 0.05) among three groups.

NBS analyses were performed among three groups to evaluate the influence of AED treatment on PSI-related functional connectivities (FC). No significant difference was found among three groups (NBS cluster size threshold > 9). If multiple comparison correction was avoided in NBS analysis, only isolated FC differences were found between AED-naïve patients and patients who underwent monotherapy (FC number = 3), and between patients who underwent mono- and poly-therapies (FC number = 3). On the other hand, more FC differences (number = 7) were found between AED-naïve patients and patients who underwent polytherapy, which formed two very small networks (**Supplementary Table S3**). This finding might suggest more, though not significant, FC variations between AED-naïve patients and patients who underwent polytherapy, possibly due to effect of AED treatment. However, since none of these differences were significant when NBS multiple comparison correction was performed, these difference might also be false positives rather than real changes. Therefore, we inferred that effect of AED treatment on PSI-related FC could be subtle in our study.

**Supplementary Table S1** Additional PSI-related FC changes in ECTS patients

| Functional connectivity | | | ECTS  (n=28) | Controls  (n=20) | *P*-value |
| --- | --- | --- | --- | --- | --- |
| Region-1 | | Region-2 |  |  |  |
| Additional subnetwork-1 | |  |  |  |  |
|  | Left Putamen | Left Pericalcarine | 0.022 | -0.123 | 0.034 |
|  | Left Putamen | Right Fusiform | -0.087 | -0.205 | 0.046 |
|  | Left Pallidum | Left Fusiform | -0.049 | -0.199 | 0.032 |
|  | Left Pallidum | Right Fusiform | -0.075 | -0.247 | 0.005 |
|  | Left Fusiform | Right Lateral Occipital | 0.348 | 0.518 | 0.010 |
|  | Left Pericalcarine | Right Pericalcarine | 1.153 | 1.369 | 0.02 |
|  | Right Entorhinal | Right Fusiform | 0.368 | 0.223 | 0.026 |
|  | Right Fusiform | Right Lateral Occipital | 0.446 | 0.622 | 0.022 |
|  | Right Lateral Occipital | Right Superior Frontal | -0.097 | -0.233 | 0.038 |
| Additional subnetwork-2 | |  |  |  |  |
|  | Left Rostral Anterior Cingulate | Right Lateral Orbitofrontal | 0.286 | 0.443 | 0.030 |
|  | Left Rostral Anterior Cingulate | Right Pars Triangularis | -0.139 | -0.001 | 0.047 |
|  | Left Superior Parietal | Right Rostral Anterior Cingulate | -0.133 | -0.267 | 0.026 |
|  | Right Lateral Orbitofrontal | Right Rostral Anterior Cingulate | 0.303 | 0.450 | 0.042 |
|  | Right Insula | Right Rostral Anterior Cingulate | 0.142 | 0.356 | 0.001 |

**Supplementary Table S2** Functional connectivity differences between ECTS patients with different seizure duration (< 24 months *vs.* ≥ 24 months)

| Functional Connectivity | | Subgroup 1  (seizure duration < 24 months) | Subgroup 2  (seizure duration ≥ 24 months) | *P*-value |
| --- | --- | --- | --- | --- |
| Region-1 | Region-2 |  |  |  |
| Left Parsopercularis | Right Inferior Temporal | 0.272 | 0.061 | 0.021 |
| Left Parsopercularis | Right Middle Temporal | 0.305 | 0.067 | 0.006 |
| Right Bankssts | Right Pars Triangularis | 0.306 | 0.015 | 0.006 |
| Right Inferior Temporal | Right Lateral Orbitofrontal | 0.253 | 0.015 | 0.044 |
| Right Middle Temporal | Right Pars Triangluaris | 0.235 | -0.087 | 0.006 |

**Supplementary Table S3**  Functional Connectivity Differences Due to AED Treatment

|  | Region-1 | Region-2 | FC | FC | *P*-value |
| --- | --- | --- | --- | --- | --- |
| AED-naïve *vs*. Monotherapy | | |  |  |  |
|  | Right Thalamus | Left Entorhinal | -0.158 | 0.079 | 0.038 |
|  | Right Bankssts | Right Superior Parietal | -0.071 | -0.245 | 0.013 |
|  | Left Postcentral | Left Precentral | 0.985 | 0.703 | 0.030 |
| AED-naïve *vs*. Polytherapy | | |  |  |  |
|  | Left Thalamus | Left Superior Parietal | -0.328 | -0.095 | 0.044 |
|  | Left Putamen | Left Pericalcarine | -0.125 | 0.081 | 0.030 |
|  | Right Thalamus | Left Temporal Pole | -0.004 | -0.206 | 0.024 |
|  | Left Pericalcarine | Right Pericalcarine | 1.384 | 1.037 | 0.032 |
|  | Left Precentral | Left Postcentral | 0.574 | 0.985 | 0.016 |
|  | Left Temporal Pole | Right Postcentral | -0.005 | 0.224 | 0.030 |
|  | Right Bankssts | Right Pars Triangularis | 0.343 | 0.061 | 0.019 |
| Monotherapy *vs*. Polytherapy | | |  |  |  |
|  | Left Precuneus | Left Transverse Temporal | -0.279 | -0.086 | 0.007 |
|  | Left Temporal Pole | Right Precentral | -0.011 | 0.195 | 0.030 |
|  | Right Isthmus Cingulate | Right Supramarginal | -0.391 | -0.190 | 0.041 |


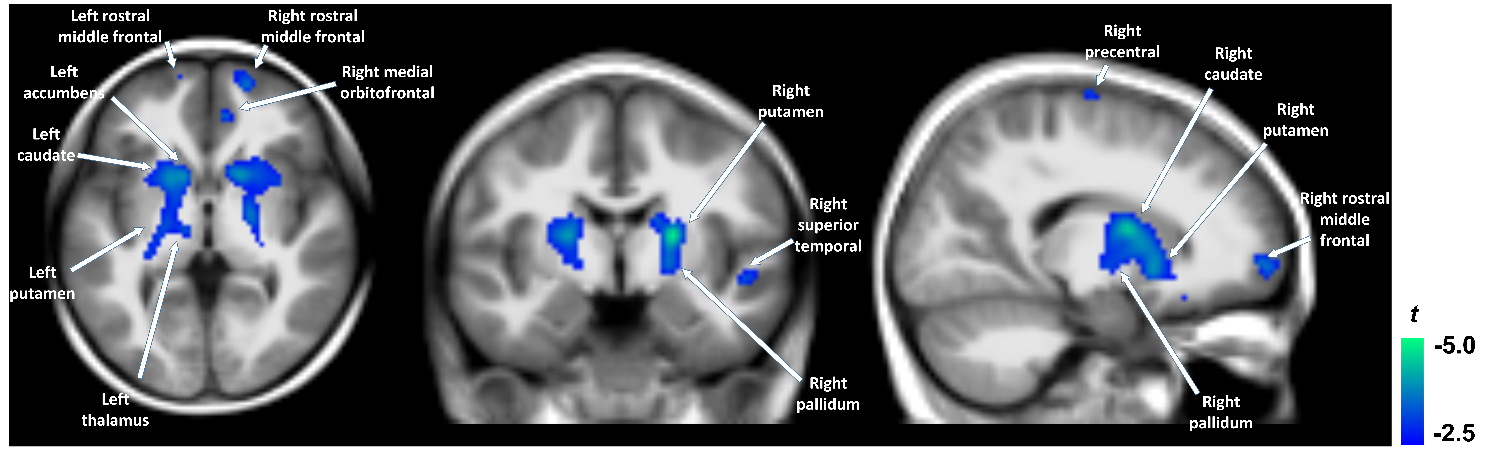


**Supplementary Figure S1.** Hypometabolism regions in patients with ECTS compared with controls.


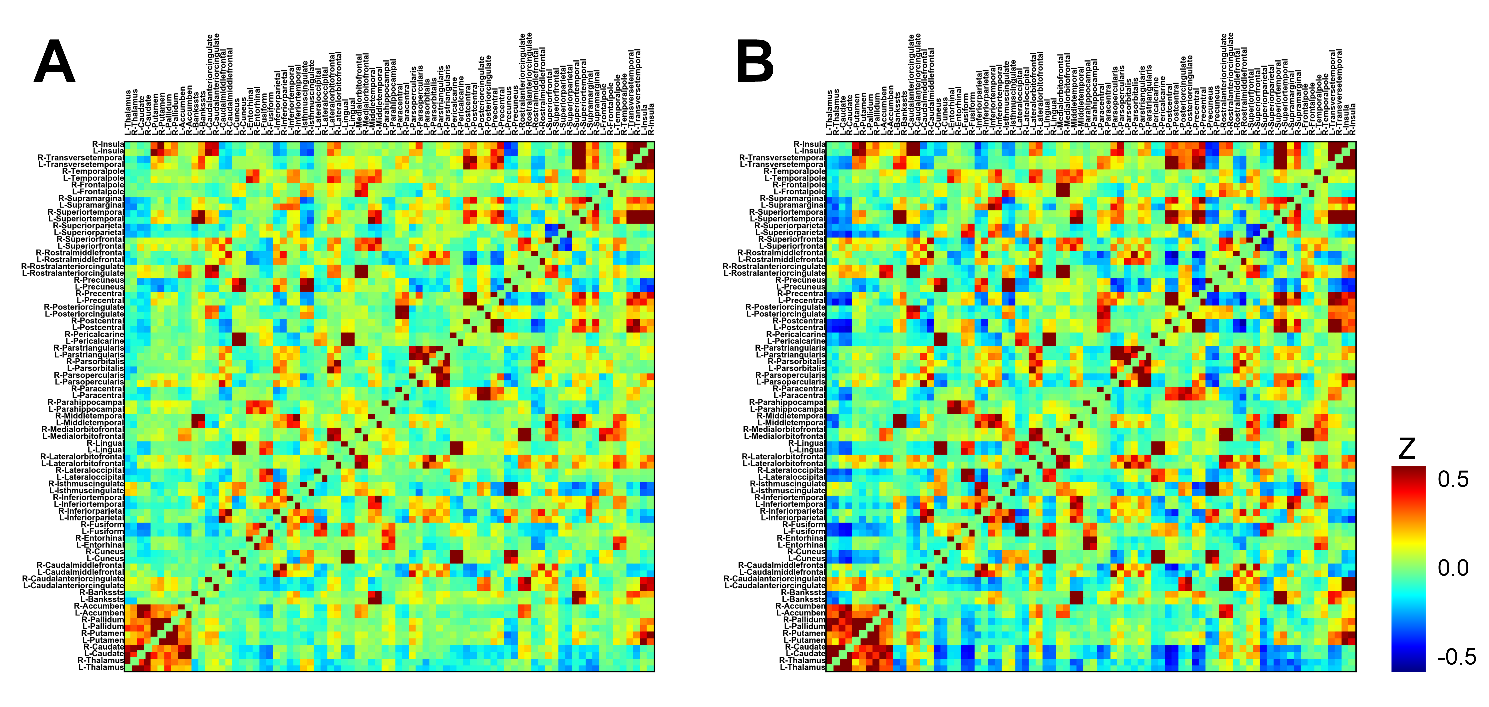


**Supplementary Figure S2:** The adjacency matrices of functional brain network related to processing speed. (A) Functional brain network of ECTS patients. (B) Functional brain network of controls. The two functional networks shared similar topology, however that of ECTS showed whole-brain functional changes compared with controls.


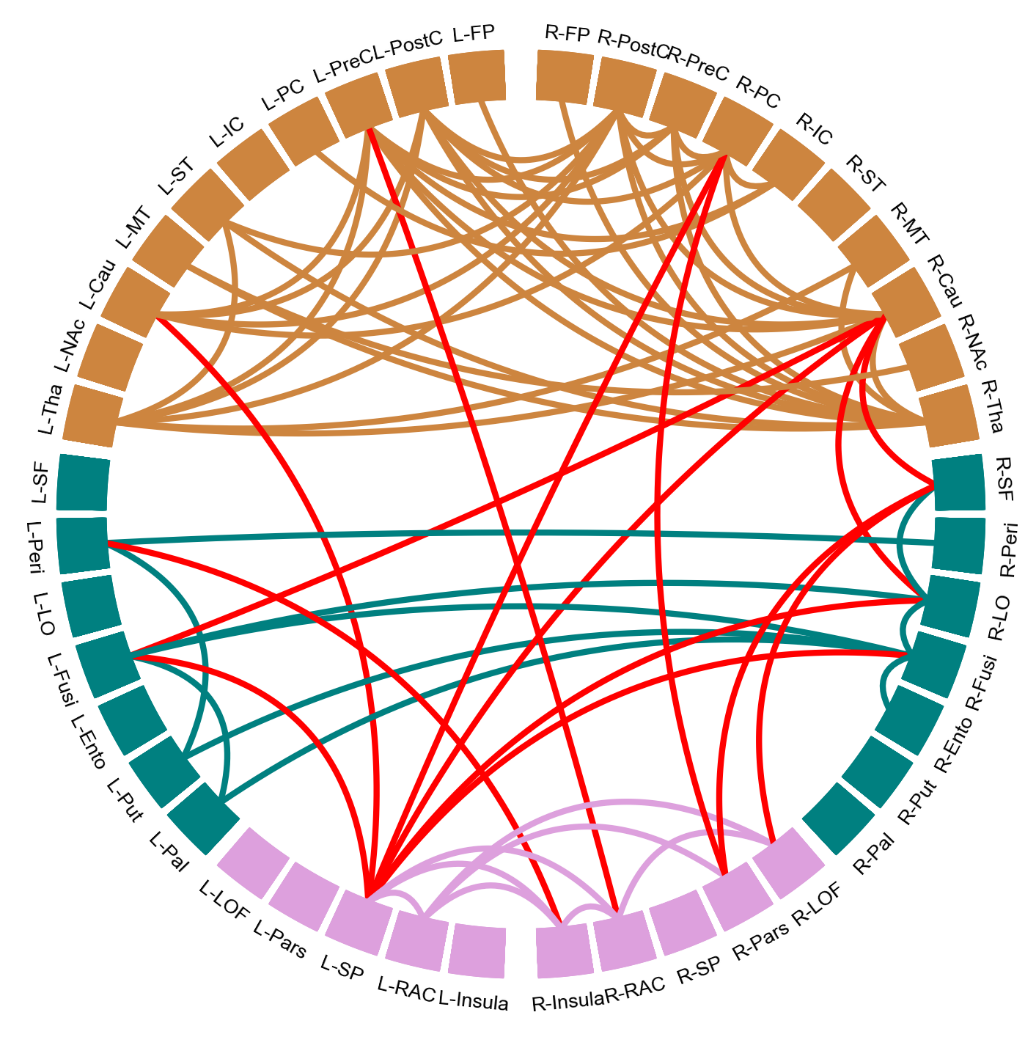


**Supplementary Figure S3** Netwok-based statatics results between ECTS patients and healthy controls when PSI-unrelated FCs were included. The red connections indicate FCs connecting different subnetworks.
